# Supplementary material for: Development of a Swellable and Floating Gastroretentive Drug Delivery System (sfGRDDS) of Ciprofloxacin Hydrochloride
Source: Pharmaceutics. 2023 May 7;15(5):1428. doi: 10.3390/pharmaceutics15051428 (PMC10223211; doi:10.3390/pharmaceutics15051428)
Supplement: Supplementary file 1 [file pharmaceutics-15-01428-s001.zip › pharmaceutics-2330936-supplementary.pdf]

Table S1. Dissolution parameters of CIP-loaded *sf*GRDDS tablets.

Groups	Formulations	Q_{2h} (%)	Q_{12h} (%)	Q_{24h} (%)	MDT [1] (h)	DE [2] (%)
SGL 60L/ HPMC 4K	F1-CIP	87.47 ± 8.86	100.54 ± 1.80	100.38 ± 0.71	0.79 ± 0.37	0.97 ± 0.01
	F2-CIP	33.88 ± 0.46	76.28 ± 1.91	95.12 ± 1.49	6.57 ± 0.48	0.69 ± 0.01
	F3-CIP	22.25 ± 0.20	60.09 ± 0.12	85.15 ± 3.77	8.34 ± 0.45	0.56 ± 0.01
SGL 60L/ HPMC 15K	F4-CIP	72.36 ± 9.68	103.03 ± 4.07	100.76 ± 1.11	1.87 ± 0.74	0.93 ± 0.02
	F5-CIP	30.20 ± 9.84	63.65 ± 6.12	90.64 ± 4.89	7.67 ± 2.50	0.61 ± 0.06
	F6-CIP	18.07 ± 1.53	54.79 ± 2.58	80.50 ± 3.66	8.85 ± 0.18	0.51 ± 0.02
SGL 90L/ HPMC 4K	F7-CIP	75.44 ± 8.54	102.43 ± 1.96	100.25 ± 0.71	1.23 ± 0.60	0.95 ± 0.02
	F8-CIP	28.49 ± 2.08	67.43 ± 10.57	90.18 ± 10.75	7.54 ± 0.78	0.62 ± 0.08
	F9-CIP	17.79 ± 0.84	54.88 ± 2.13	80.21 ± 0.74	8.61 ± 0.42	0.51 ± 0.02
SGL 90L/ HPMC 15K	F10-CIP	64.14 ± 9.33	100.76 ± 2.89	101.29 ± 1.72	2.35 ± 0.64	0.91 ± 0.03
	F11-CIP	22.31 ± 0.79	62.52 ± 3.55	85.94 ± 3.11	8.01 ± 0.81	0.57 ± 0.01
	F12-CIP	17.24 ± 0.31	53.33 ± 1.95	80.45 ± 1.48	9.10 ± 0.16	0.50 ± 0.01
HPMC 4K	F13-CIP	56.44 ± 4.62	102.62 ± 2.09	103.83 ± 2.87	2.47 ± 0.46	0.93 ± 0.01
	F14-CIP	23.46 ± 1.80	64.73 ± 4.71	95.04 ± 5.63	8.67 ± 1.29	0.61 ± 0.02
	F15-CIP	17.39 ± 0.64	59.23 ± 0.57	91.49 ± 6.95	9.34 ± 0.61	0.56 ± 0.02
HPMC 15K	F16-CIP	36.88 ± 0.34	89.33 ± 9.55	101.54 ± 6.60	5.07 ± 1.24	0.80 ± 0.02
	F17-CIP	17.51 ± 1.82	60.80 ± 6.74	88.12 ± 0.45	8.85 ± 1.02	0.56 ± 0.04
	F18-CIP	15.71 ± 0.27	56.14 ± 4.73	84.66 ± 6.20	9.30 ± 0.10	0.52 ± 0.04
MCC	F19-CIP	100.01 ± 5.33	104.10 ± 1.84	105.06 ± 0.81	0.76 ± 0.30	1.02 ± 0.01
	F20-CIP	90.51 ± 4.25	103.55 ± 1.83	105.35 ± 2.33	1.32 ± 0.66	1.00 ± 0.01
	F21-CIP	93.37 ± 9.77	102.94 ± 0.90	102.43 ± 1.69	0.90 ± 0.68	0.99 ± 0.02
Commercial tablets	Ciproxin®	104.38 ± 1.40	104.07 ± 0.77	104.01 ± 1.11	0.48 ± 0.07	1.02 ± 0.01

[1] MDT = mean dissolution time; [2] DE = dissolution efficiency. Data represent mean ± standard error of the mean (n = 3 for each group).
